# Diagnostic, Prognostic, and Recurrence Monitoring Value of Plasma CYFRA21-1 and NSE Levels in Patients With Esophageal Squamous Cell Carcinoma

**DOI:** 10.3389/fonc.2021.789312

**Published:** 2022-01-21

**Authors:** Mengyang Ju, Xiaolin Ge, Xiaoke Di, Yumeng Zhang, Liang Liang, Yujing Shi

**Affiliations:** ^1^ Department of Radiation Oncology, Osaka University Graduate School of Medicine, Osaka, Japan; ^2^ Department of Radiotherapy, Jiangsu Provincial People’s Hospital, Nanjing, China; ^3^ Department of Oncology, Graduate School of Nanjing Medical University, Nanjing, China; ^4^ Department of Oncology, The People’s Hospital of Jurong City, Zhenjiang, China

**Keywords:** CYFRA21-1, NSE, ESCC, diagnosis, prognosis, recurrence monitoring

## Abstract

This study was aimed to evaluate the clinical values of single markers and combination in the diagnosis, short-term efficacy and recurrence risk assessment of esophageal squamous cell carcinoma (ESCC).

**Methods:**

Totally 50 patients with I-IVa stage ESCC, 50 healthy controls and 11 patients with recurrent esophageal cancer after comprehensive treatment were enrolled. Serum biomarkers were collected and evaluated. Serum concentrations of carcinoembryonic antigen (CEA), cytokeratin 19 fragment antigen 21-1 (CYFRA21-1) and neuron specific enolase (NSE) were measured by enzyme-linked immunosorbent assay before and after treatment.

**Results:**

The diagnostic efficacy ROC curve area of CEA, CYFRA21-1 and NSE in esophageal cancer was 0.70, 0.71 and 0.64(all P <0.05), respectively, the sensitivity was 80%, 88.89% and 60% respectively, and the specificity was 53%, 58.5% and 58% respectively. The sensitivity and specificity of the combined detection were 68% and 78% respectively. The area under ROC curve was 0.75. CEA, CYFRA21-1 and NSE were significantly higher than the healthy control group and thus can be used as diagnostic markers of esophageal cancer (all P <0.05). After standard treatment, the clinical CR and PR rate of patients with positive CYFRA21-1 or NSE before treatment was significantly lower than that of patients with negative CYFRA21-1 or NSE (X^2 =^ 4.52,P =0.03). A significant negative correlation was found between N stage and clinical efficacy (HR 2.48, 95%CI 1.07-5.73). After comprehensive treatment, the serum CYFRA21-1 and NSE levels in recurrent patients also increased significantly(all P<0.05), indicating these two markers play obvious roles in recurrence monitoring.

**Conclusion:**

CYFRA21-1 and NSE may help to predict the response of ESCC to CRT, and play important roles in the diagnosis and recurrence monitoring of esophageal cancer. These markers have a diagnostic value of esophageal cancer when combined with CEA.

## Introduction

The incidence and mortality rates of esophageal cancer, one common gastrointestinal tumor, rank seventh and sixth respectively among all malignant tumors, according to a global cancer report (1). These two rates increase year by year, and the 5-year survival rate of esophageal cancer is less than 20%, according to data of sexually transmitted diseases. Compared with developed countries, the incidence and mortality of esophageal cancer in developing countries are higher. Especially, the incidence rate and mortality of this disease are the highest in China, accounting for 54.1% of total cases and 56% respectively (2). Esophageal squamous cell carcinoma (ESCC) and esophageal adenocarcinoma are two major pathological types. ESCC accounts for more than 90% of esophageal cancer patients in China. ESCC usually presents with occult attack, high degree of malignancy, and rapid progression. Therefore, most patients with ESCC are at the middle and advanced stages, resulting in poor treatment effect and poor prognosis. At present, surgery, radiotherapy and chemotherapy are the main treatment methods of ESCC, but most patients have lost the opportunity of surgery. Radical radiotherapy and chemotherapy are used as the main treatment methods of locally advanced ESCC. Some esophageal cancer patients who have reached CR or PR after comprehensive treatment still suffer recurrence within 1 year (1). The heterogeneity of prognosis suggests it particularly important to evaluate the risk of patients and find reliable evaluation indicators. Use of imaging methods is still a challenge for the diagnosis and efficacy evaluation of ESCC. Therefore, in clinical practice, finding non-invasive detection methods to evaluate the treatment effect, monitor tumor recurrence, and even screen out high-risk recurrence groups is urgent.

Tumor markers are substances produced by the synthesis and release of tumors or by the stimulation response of tumor cells to the body. They have certain specificity for various tumors and are widely used in the diagnosis and treatment of some tumors. It is considered that the increase of alpha feto protein (AFP) level is closely related to liver cancer, and the significant rise of carcinoembryonic antigen (CEA) level is linked with digestive system tumors. Similarly, significant elevation of CA153 level, and CA125/CA72-4 levels are associated with breast cancer and ovarian cancer respectively. Pancreatic cancer is more frequent when chain antigen 19-9 (CA19-9) level is elevated. Finally, when neuron specific enolase (NSE) level significantly elevates, it often indicates neuroendocrine tumors or small cell lung cancer. However, there are few studies on tumor markers specific for esophageal cancer. Reportedly, cytokeratin protein fragment 19 (CYFRA21-1), CEA and sugar CA19-9 are commonly-used tumor markers of ESCC, and can help to judge the curative effect and prognosis of ESCC (3, 4), but are all limited by low specificity. In addition, few studies involve the changes of tumor markers in peripheral blood of esophageal cancer patients before and after treatment, or before and after radical radiotherapy and chemotherapy.

We included 50 ESCC patients who underwent radical radiotherapy and chemotherapy in the treatment center to detect tumor markers in peripheral blood, mainly including AFP, CEA, CA19-9, CA72-4, NSE and CYFRA21-1. By evaluating the dynamic changes of these tumor markers before and after radiotherapy and chemotherapy, we analyzed the relationship between the positive rate of tumor markers and clinicopathological factors. Then the relationship between tumor marker concentrations and clinical efficacy was analyzed. At the same time, the changes of tumor markers in the serum of a healthy control group and a recurrent esophageal cancer group were evaluated to further clarify the roles of tumor markers in the diagnosis and recurrence monitoring of esophageal cancer.

## Methods

### Case Data

The 50 ESCC patients underwent radical radiotherapy and chemotherapy between March 2018 and December 2020, including 43 males and 7 females. Their ages ranged from 47 to 81 years, with an average of 67.06 years. At the same time, 11 patients with recurrent esophageal cancer were included, including 10 males and 1 female, who were aged 48-75 years (mean 64.9 years). A healthy control group including 25 males and 25 females was set, who were aged 29-83 years (mean 55.6 years).

Inclusion criteria were: (1) esophageal cancer confirmed as ESCC by histopathological biopsy; (2) All no reception of any treatment before radiotherapy and chemotherapy; (3) no cognitive impairment and ability of normal communication. The healthy controls had no malignant tumor, benign tumor or other benign diseases. Exclusion criteria were: (1) abnormal liver and kidney function; (2) immune and nervous system disorders; (3) serious noncooperation and withdrawal from the researcher; (4) other malignancies. All patients signed informed consent before treatment.

### Experimental Methods

#### Grouping

Peripheral blood samples of the 50 ESCC patients undergoing radical radiotherapy and chemotherapy within 1 week before treatment and 1 month after treatment were collected and recorded as Pretreatment and Posttreatment Groups respectively. At the same time, the peripheral blood of recurrent esophageal cancer patients within 1 week before treatment was collected and recorded as a Recurrence Group. Similarly, peripheral blood samples from a number of 50 healthy people at the same time were collected and recorded as Healthy Controls. The changes of tumor markers in peripheral blood samples of each group before and after treatment were analyzed to clarify the types of markers with obvious changes before and after treatment, and to further clarify the types of markers for efficacy evaluation in esophageal cancer treatment.

#### Detection of Tumor Markers

The concentrations of serum AFP, CEA, CA72-4, CYFRA21-1, CA19-9 and NSE were detected by enzyme-linked immunosorbent assay (ELISA) using an Au5800 automatic biochemical instrument (Beckman Kurt company, USA). Specifically, 5 ml of fasting elbow venous blood was extracted, centrifuged at 3500 r/min for 20 min, and then the upper serum was taken and put into a - 70°C low-temperature refrigerator for use. The instrument and detection kits (Shanghai Taikang Biotechnology Co., Ltd.) were operated in strict accordance with the instructions. According to the instructions of the kits and the data of our institution, the cut-off values of the tumor markers were set as AFP < 20 ng/ml, CEA < 4.7 ng/ml, CA19-9 < 39 u/ml, CA72-4 < 6.9 u/ml, CYFRA21-1 < 3.3 ng/ml and NSE < 16.3 ng/ml. If a marker is greater than the corresponding cut-off value, it is considered positive.

#### Observation Indexes

(1) Serum concentrations of the five tumor markers were compared. (2) Diagnostic values of the five tumor markers in esophageal cancer were analyzed. (3) Relationship between the tumor marker concentrations and clinicopathological features in the pretreatment group was tested. (4) Relationship between the positive rate of serum tumor markers before treatment and the short-term clinical efficacy was studied to further clarify the types of tumor markers with monitoring values. (5) Imaging evaluation: chest and abdomen enhanced computed tomography (CT) or positron emission tomography (PET)-CT was performed 1 month after radical radiotherapy and chemotherapy to evaluate the curative effect.

The four Recist1.1 evaluation criteria were adopted.

Complete response (CR): all target lesions disappeared completely, and the short axis value of lymph nodes was < 15 mm.

Partial response (PR): the sum of ‘the maximum length and diameter of the target lesions’ continually decreased by more than 30% within 4 weeks.

Disease progression (PD): the above sum increased by more than 20.0%, or new lesions appeared.

Stable disease (SD): the sum decreased by less than 30.0% or increased by less than 20.0%.

CR and PR indicate ‘effective’, but SD and PD suggest ‘ineffective’.

#### Treatment Plan

Intensity-modulated radiation therapy was selected as the radiotherapy technology. The prescribed dose of radiotherapy was 50-66 Gy/25-33f/1.8-2 Gy. The treatment plan was 95% PTV/PGTV/PGTVnd get 50-66Gy irradiation dose, and the limits of endangering organs, lungs, heart and spinal cord were within the normal ranges. The chemotherapy regimen was: one cycle every 21 days, a total of two cycles of chemotherapy during radiotherapy. The specific chemotherapy regimen was paclitaxel liposome 135 mg/m^2^ combined with 100 mg/m^2^ nedaplatin.

### Statistical Processing

Statistical analysis of data was conducted Graphpad 8.0.2. The measured data were expressed as (x ± s). The measured data that did not conform to the normal distribution were displayed as the median. The changes of tumor markers before and after treatment were evaluated by Kruskal Wallis nonparametric rank sum test. Inter-group and multigroup comparisons were examined by Mann Whitney u test and by one-way analysis of variance (ANOVA) or Chi-square test respectively. Relationship between the changes of tumor markers and clinicopathological factors was tested by logistic regression analysis. The differences were significant at P < 0.05.

## Results

### Patient Situation

The 50 esophageal cancer patients receiving radical radiotherapy and chemotherapy all completed the treatment, and 100 peripheral blood samples were obtained before and after treatment. At the same time, 50 peripheral blood samples from the control group and 11 samples from the recurrent group were acquired. The 50 esophageal cancer patients were divided into four clinical stages, including 2, 19, 18 and 11 patients at stages I, II, III and IVA respectively, according to the 7th edition of the American Joint Commission on cancer (AJCC). The tumors were located in the cervical segment in 10 cases, the thoracic segment in 24 cases and the lower thoracic segment in 16 cases. There were 10, 1, 4, 3, 31 and 1 patient receiving 50 Gy, 56 Gy, 58 Gy, 59.92 Gy, 60 Gy and 66 Gy doses respectively. All patients with radical radiotherapy and chemotherapy underwent imaging examination to evaluate their conditions one month after treatment, mainly including chest and abdomen enhanced CT or PET-CT. The examination results were independently evaluated by two radiologists & imaging doctors with senior years or above. When the evaluation results of the two evaluators were inconsistent, they decided after discussion. After radical radiotherapy and chemotherapy according to Recist1.1 evaluation standard, 4, 26, 14 and 6 cases reached clinical CR, PR, SD, and PD respectively (**Table 1**).

**Table 1:** Clinical pathology data of patients with esophageal cancer.

Characteristic	Number of Patients (%)
Age (years)	
Median Age	67.06
Range	47-83
Gender	
Male	43 (86%)
Female	7 (14%)
Tumor Location	
Upper	10 (20%)
Middle	24 (48%)
Lower	16 (32%)
ECOG	
0-1	46 (92%)
2	4 (8%)
DT (Gy)	
50	10 (20%)
56	1 (2%)
58	4 (8%)
59.92	3 (6%)
60	31 (62%)
66	1 (2%)
pTNM Stage	
I	2 (4%)
II	19 (38%)
III	18 (36%)
IV	11 (22%)
Pos-CCRT	
SD	14 (28%)
PR	26 (52%)
CR	4 (8%)
PD	6 (12%)

DT, dose total; Pos-CCRT, Post-Concurrent chemoradiotherapy.

### Plasma Tumor Markers Levels in All the Samples

First, we analyzed the serum concentrations of AFP, CEA, CA72-4, CYFRA21-1, CA19-9 and NSE in each group. The positive detection rates of tumor markers in each group were calculated according to the reference values of all reagents. Results showed AFP, CA72-4 and CA19-9 concentrations in the pretreatment and control groups. The positive rates in the controls were very low (**Table 2**).

**Table 2:** Positive rate of tumor markers in each group.

Tumor Marker	Reference Value	Pretreatment Group	Posttreament Group	Recurrence Group	Healthy Controls
AFP	<20ng/mL	0 (0/50)	0 (0/50)	0 (0/11)	0 (0/50)
CEA	<4.7ng/mL	16% (8/50)	12% (6/50)	45% (5/11)	4% (2/50)
CA19-9	<39 U/mL	2% (1/50)	0 (0/50)	36% (4/11)	0 (0/50)
CA72-4	<6.9 U/mL	2% (1/50)	16% (8/50)	18% (2/11)	4% (2/50)
CYFRA21-1	<3.3 ng/mL	34% (17/50)	8% (4/50)	64% (7/11)	4% (2/50)
NSE	<16.3ng/mL	58% (29/50)	42% (21/50)	81.8% (9/11)	42% (21/50)

### Diagnostic Value of Plasma Tumor Markers Levels in Patients With ESCC

**Figure 1:**
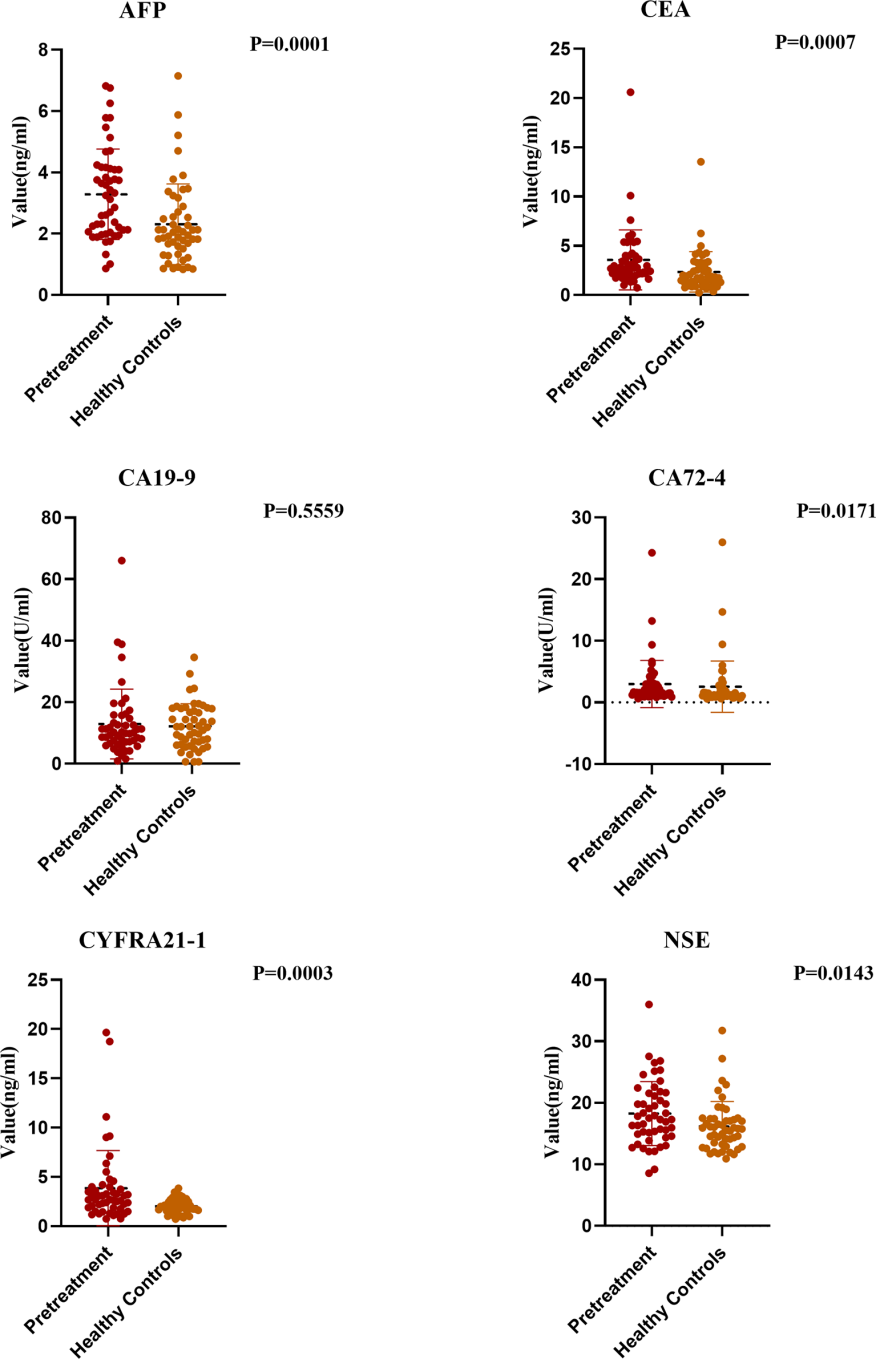
Comparison of Tumor Markers between Pretreatment Group and Healthy Controls.

To determine whether the tested tumor markers are valuable for diagnosis of esophageal cancer, we analyzed the data of the pretreatment group and the control group. No significant difference was found in the CA19-9 concentration between the two groups, and levels of the other tumor markers were higher in the pretreatment group than in the control group. Although the levels of AFP and CA72-4 were significantly different between the two groups (both P < 0.05), considering the very low positive detection rates of the two groups, we believe they had no diagnostic values for ESCC and thus did not further study. When the cut-off value of CEA was 4.7 ng/ml, the fluctuation range of CEA was 2.35 ± 2.0 ng/ml in the control group and was 3.5 ± 3.03 ng/ml in the pretreatment group, which were significantly lower than in the esophageal cancer group (P < 0.05). The fluctuation range of CYFRA21-1 was 1.9 ± 0.74 ng/ml in the healthy control group and was 3.85 ± 3.8 ng/ml in the pretreatment group, which were significantly lower than in the esophageal cancer group (P < 0.05). Similarly, when the cut-off value of NSE was 16.3 ng/ml, the change range of NSE was 16.1 ± 4 ng/ml in the control group and was 18.2 ± 5.2 ng/ml in the pretreatment group, with significant difference (P < 0.05) (**Figure 1**).

**Figure 2:**
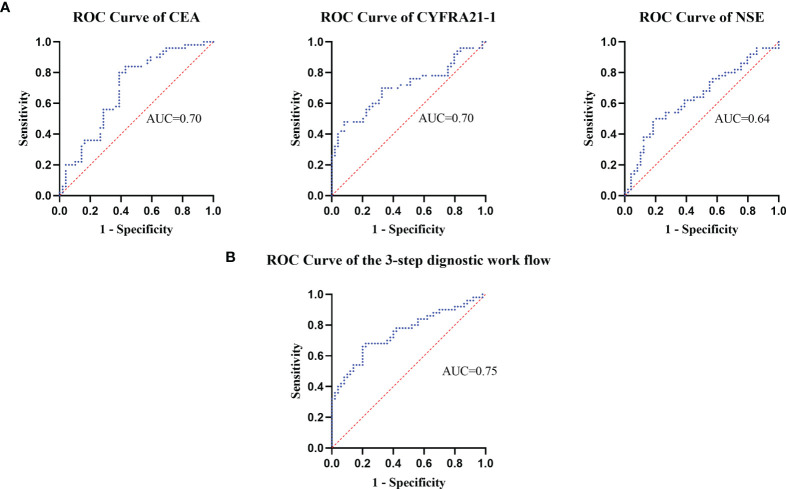
The receiver operating curves were used to assess the dignostic value of plasma tumor marker's levels.

The receiver’s operating curves (ROCs) of CEA, CYFRA21-1 and NSE in the diagnostic efficacy of ESCC were further studied. The areas under the curves of CEA, CYFRA21-1 and NSE were 0.70, 0.71 and 0.64 respectively (all P < 0.05), the sensitivity was 80%, 88.89% and 60% respectively, and the specificity was 53%, 58.5% and 58% respectively, indicating that the above three tumor markers have obvious diagnostic values in esophageal carcinoma, especially CYFRA21-1. Interestingly, when the cut-off value of NSE was set at 16.3 ng/ml, the positive rate in the control group was up to 42%. Therefore, analysis of the detection value of NSE in ESCC found that when the cut-off value of NSE was 17.5 ng/ml, the Yoden index was 0.40. At this time, the diagnostic efficiency of NSE is the highest, with diagnostic sensitivity of 50% and specificity of 87.6% (**Figure 2A**).

**Figure 3:**
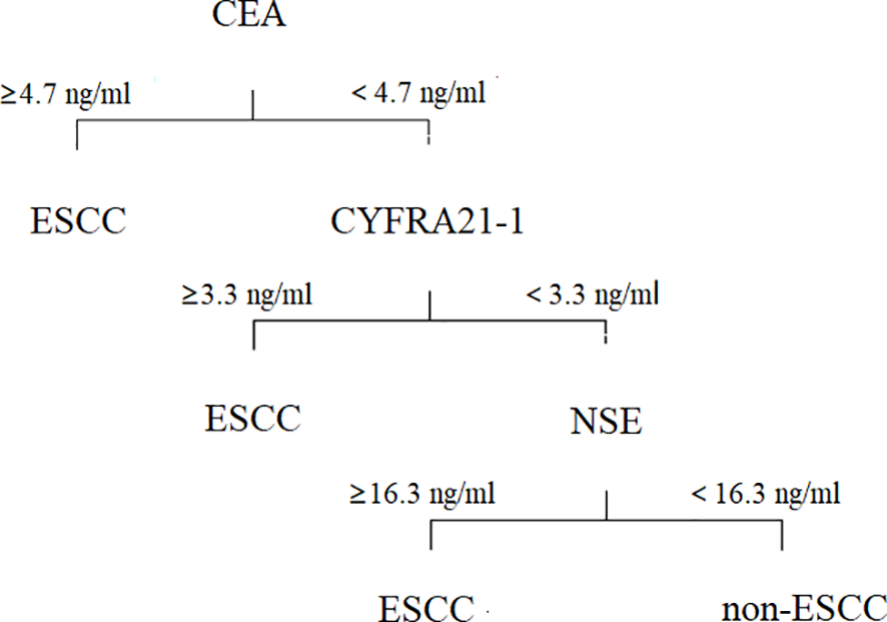
A 3-step diagnostic work flow based on tumor markers for patients in ESCC.

Obviously, it is not a proper choice to use a single positive tumor marker for diagnosis and prognosis. To identify an effective diagnostic profile with an improved discriminative accuracy between ESCC and non-ESCC patients, we set a 3-step diagnostic procedure. First, plasma levels of CEA were taken into account. If such levels were found to be equal or above the detection threshold of 4.7 ng/mL, the diagnosis of ESCC was retained. On the contrary, if plasma CEA levels were negative, the diagnosis relied on the plasma concentration of CYFRA21-1, which accounted for the detection threshold of 3.3 ng/mL. At last, with the threshold of 16.3 ng/ml, plasma levels of NSE played a significant diagnostic role if both CEA and CYFRA21-1 levels were normal (**Figure 3**).

The receiver operator characteristic analysis accounting for the 3-step diagnostic procedure afforded an area under the curve of 0.75 for the diagnosis of patients with ESCC, with a sensitivity of 68% and a specificity of 78% (**Figure 2B**).

### Prognostic Value of Plasma Tumor Markers Levels in Patients With ESCC

**Figure 4:**
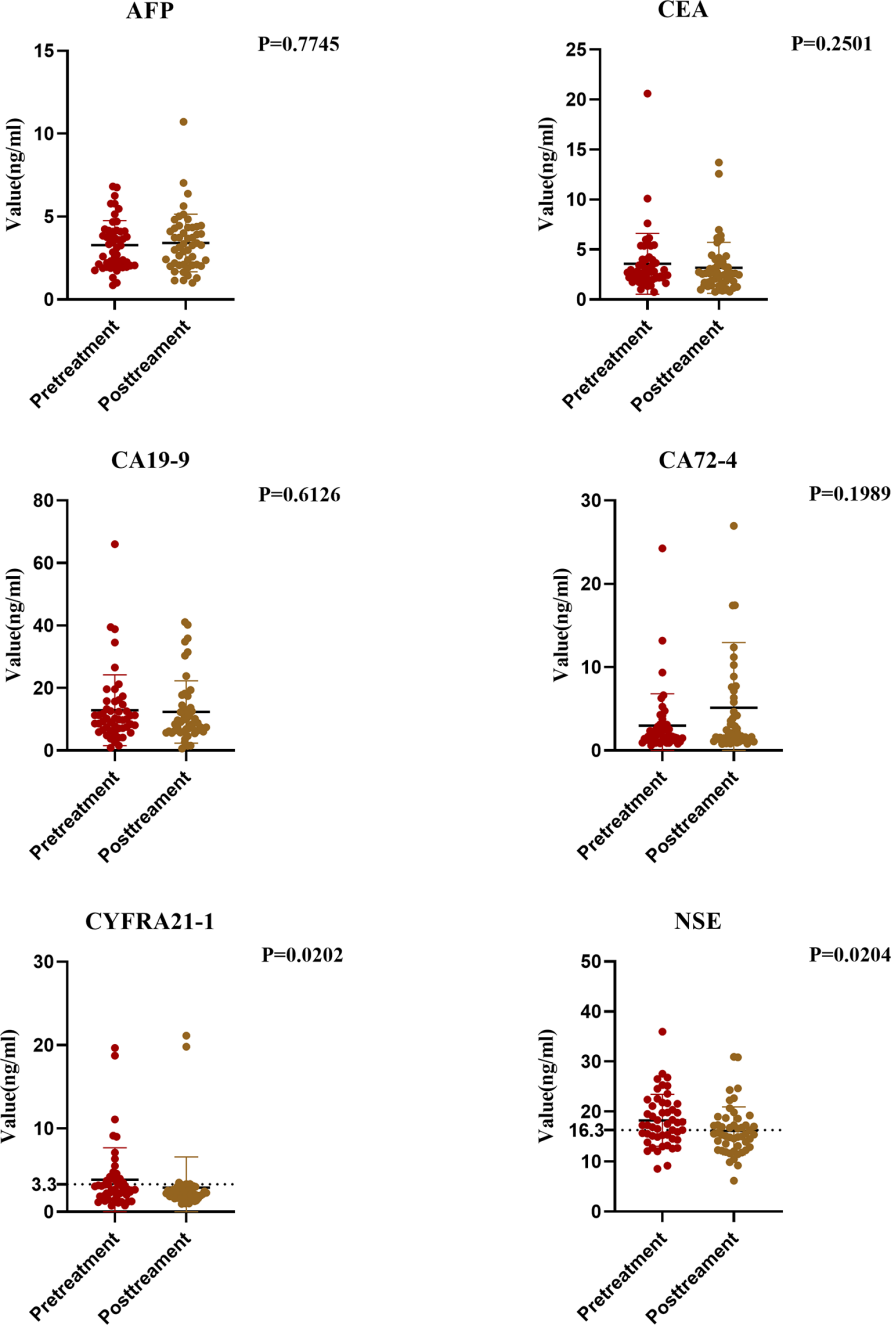
Comparison of Tumor Markers between Pretreatment and Posttreatment Group.

We compared the data of the pretreatment group and the posttreatment group. Results showed significant differences in the changes of tumor markers before and after treatment. The serum CA72-4 level in the posttreatment group was higher than that in the pretreatment group, but not significantly (P > 0.05). The serum levels of CEA, CA-199, CYFRA21-1 and NSE in the posttreatment group were significantly lower, and the decrease of CYFRA21-1 and NSE was significant (P < 0.05) (**Figure 4**).

Therefore, we studied the relationship between the two significant markers and clinicopathological factors. When the cut-off value of CYFRA21-1 was set at 3.3 ng/ml, the positive rate of CYFRA21-1 before treatment was significantly correlated with patient gender, tumor location, T stage, N stage, and tumor, node, metastasis (TNM) stage, but not with age or body mass index (BMI) (P > 0.05). The positive rate at a later stage was higher (all P < 0.05). Similarly, the diagnostic efficiency was the highest when the cut-off value of NSE was 17.5 ng/ml. Therefore, we studied the cut-off value of NSE at 17.5 ng/ml and found the positive rate of NSE before treatment was not significantly correlated with age, gender, BMI, T stage, N stage, TNM stage or tumor location (all P > 0.05) (**Table 3**).

**Table 3:** Assessment of plasma tumor markers’ levels by multivariate analysis.

Characteristic	Levels of CYFRA21-1 (ng/ml)		X^2^	P-Value	Levels of NSE (ng/ml)		X^2^	P-Value
	<3.3	≥3.3			<17.5	≥17.5		
Age (Years)			2.71^a^	0.12			0.001^a^	1
<65	10	3			7	18		
≥65	23	4			11	14		
Gender			8.05^b^	0.005			1.78^b^	0.32
M	28	15			21	22		
F	6	1			4	3		
BMI			0.16^b^	0.8			1^a^	0.31
<24	22	12			15	16		
≥24	12	4			10	9		
T-Stage			8.66^b^	0.003			1.4^b^	0.78
T1	2	0			2	0		
T2	9	1			7	5		
T3	20	11			13	17		
T4	2	4			3	3		
N-Stage			9.09^b^	0.026			1.45^b^	0.73
N0	17	4			9	12		
N1	9	6			3	12		
N2	4	2			4	3		
N3	1	3			2	3		
AJCC-Grade			12.25^b^	0.003			0.74^b^	0.39
I-II	18	3			12	9		
III-IV	14	15			9	20		
Location			13.21^b^	0.001			2.31^b^	0.32
Upper	10	0			4	6		
Middle	14	10			15	9		
Lower	9	7			7	9		

^a^Chi square test; ^b^Fisher exact test.

**Figure 5:**
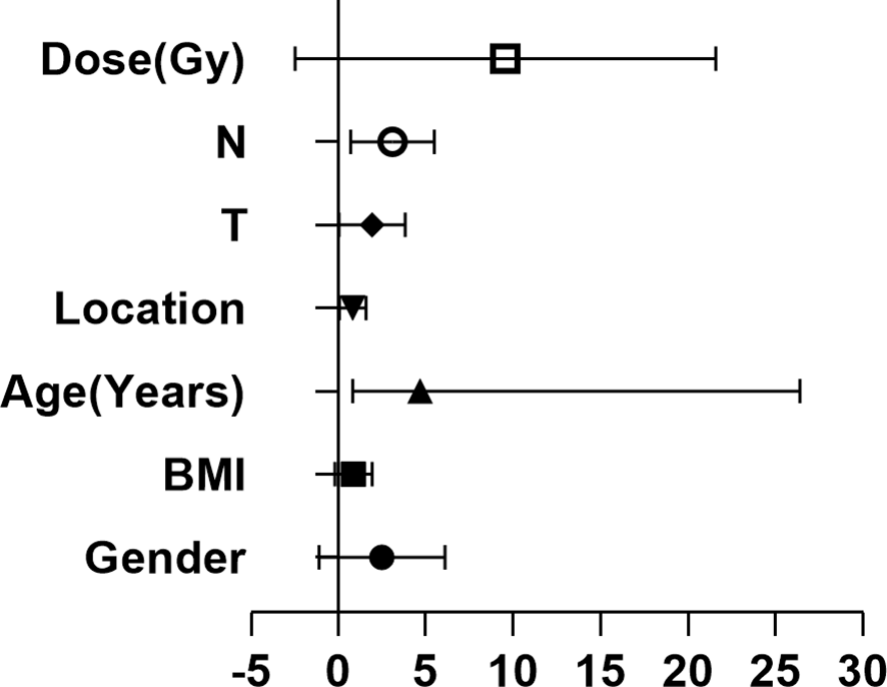
Relationship between clinical parameters and efficacy.

To further clarify the relationship of clinical efficacy with the positive rates of NSE and CYFRA21-1 before treatment, we recorded as tumor maker positive TM(+) when neither NSE or CYFRA21-1 was positive before treatment, and as TM(-) when both were negative. Before radical radiotherapy and chemotherapy, the TM(+) rate was 64% (32/50), and the TM(-) rate was 36% (18/50). After radiotherapy and chemotherapy, the clinical CR + PR rate was 46.9% (15/32) in the TM (+) patients and 72.2% (13/18) in the TM (-) patients. The positive rate of tumor markers before treatment significantly and directly affected the CR + PR rate (x2 = 4.52, P = 0.03). Thus, pretreatment TM(-) patients benefited more from the treatment and were more likely to achieve clinical CR or PR. In addition, the serum TM level of esophageal cancer patients was significantly decreased after treatment (P = 0.03). We then analyzed the clinicopathological factors affecting the short-term efficacy through logistic regression. It was found that none of gender, age, BMI, tumor location, T stage or radiotherapy dose was significantly correlated with the short-term efficacy of radiotherapy and chemotherapy, while the main influence factor on the efficacy of was lymph node metastasis (P = 0.03, HR 2.48, 95%CI 1.07-5.73). At the same time, we studied 20 patients who were determined to be ineffective after radiotherapy and chemotherapy. The rates of TM(+) and TM(-) were 80% (16/20) and 20% (4/20), respectively, and 50% (10/20) and 50% (10/20) respectively after treatment (**Table 4** and **Figure 5**).

**Table 4:** Logistic regression analysis of the relationships between effectiveness of chemoradiotherapy (CRT) and serum levels of CYFRA21-1 and NSE.

Characteristic	P-Value	Hazard ratio	95% Confidence interval	
			Lower	Upper
Gender	0.79	0.75	0.08	6.63
BMI	0.28	0.41	0.08	2.07
Age (Years)	0.08	4.68	0.83	26.4
Location	0.32	0.55	0.21	1.67
T	0.64	1.31	0.42	4.04
N	0.03	2.48	1.07	5.73
Dose (Gy)	0.07	4.5	0.86	23.3

### Recurrence Monitoring Value of Plasma Tumor Markers Levels in Patients With ESCC

**Figure 6:**
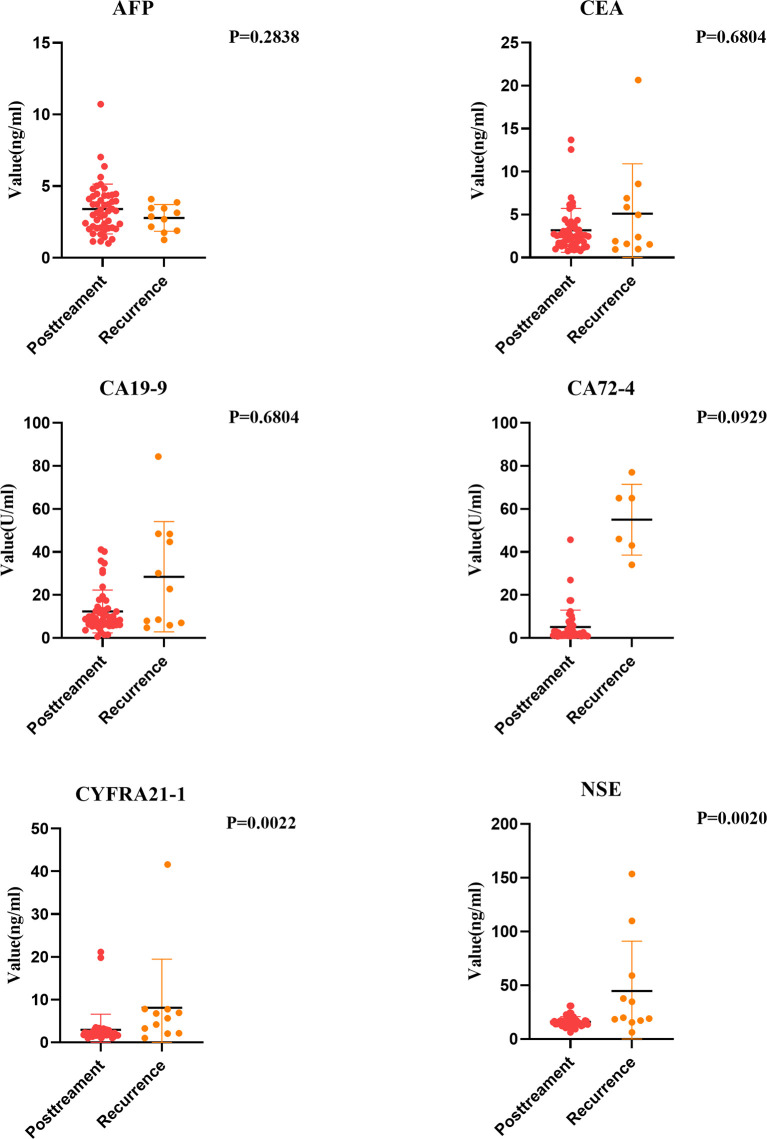
Comparison of Tumor Markers between Pretreatment and Recurrence Group.

Some studies point out that some esophageal cancer patients will suffer tumor recurrence within one year after comprehensive treatment. Therefore, it is very important to find out tumor markers that can help monitor the risk of recurrence. We analyzed the tumor markers in the recurrent group and the posttreatment group. Results showed that except AFP, the positive rates of other tumor markers in the serum of esophageal cancer patients in the recurrent group increased to varying degrees. NSE and CYFRA21-1 levels increased significantly (P < 0.05). Moreover, regular examination of CYFRA21-1 and NSE in esophageal cancer patients after treatment can effectively monitor recurrence. If CYFRA21-1 and NSE levels elevated, the patient should be highly vigilant against the risk of tumor recurrence (**Figure 6**).

## Discussion

ESCC has a hidden onset and no obvious symptoms in early stage. It is often diagnosed at the middle and late stage and thus loses the opportunity of operation. Radical radiotherapy and chemotherapy become the primary treatments for esophageal cancer. However, radiotherapy shows great individual differences. The clinical effects of radiotherapy on ESCC patients with similar or the same pathological stages are different. At present, CT is often used for efficacy evaluation. However, based on radiation and delay, CT is unsuitable for repeated use as efficacy evaluation (5). Therefore, selecting non-invasive and highly repetitive methods that can effectively evaluate the short-term efficacy and recurrence monitoring is very important. Reportedly, tumor markers are potential for screening, diagnosis, prognosis establishment, treatment monitoring and recurrence detection (6). However, it is unclear whether the pretreatment serum levels of tumor markers are important predictors of prognosis and whether clinicopathological factors are independent of the TNM staging system, for esophageal cancer patients with concurrent radiotherapy and chemotherapy.

Through detecting tumor markers in peripheral blood of 50 ESCC patients before and after radiotherapy and chemotherapy, we found the sensitivity of tumor markers before treatment was 0% (AFP), 0% (CA19-9), 33% (CA72-4), 80% (CEA), 88.9% (CYFRA21-1) and 60% (NSE). Especially, AFP, CA19-9 and CA72-4 all had low sensitivity in diagnosis of esophageal cancer, which is consistent with previous studies (7–9). Further research found no significant difference in the changes of AFP, CA19-9 or CA72-4 in peripheral blood after treatment. Therefore, we believe these marks have no significant value in monitoring the short-term efficacy, diagnosis and treatment of esophageal cancer, which were not further studied. CEA was first isolated from colon cancer in 1965, and its diagnostic and prognostic roles in esophageal cancer were confirmed (9). However, its diagnostic and monitoring values after concurrent radiotherapy and chemotherapy were rarely studied. In this study, the diagnostic sensitivity of CEA for esophageal cancer is up to 80%, higher than those of AFP, CA19-9 and CA72-4. The high diagnostic value of CEA in esophageal cancer is consistent with a previous study (10). After further comparing the CEA levels among the pretreatment group, recurrent group and posttreatment group, we found no significant changes in CEA after treatment (P > 0.05), and no significant difference in serum CEA between the posttreatment group and the recurrence group (P > 0.05). Therefore, we believe CEA plays a poor role in the prediction of short-term curative effect and recurrence monitoring after treatment, which is consistent with a previous study (11).

Reportedly, CYFRA21-1 is produced by tumor cells. In a cancerous body, tumor cells can release CYFRA21-1 soluble fragments into the blood circulation (12), and its numerical change is positively correlated with tumor size and disease progression (13). CYFRA21-1 was first found as a tumor marker of lung cancer, and its role in the diagnosis and monitoring of lung cancer and head and neck squamous cell carcinoma was confirmed (13–15). Recently, the role of CYFRA21-1 in evaluating the efficacy of esophageal cancer after chemotherapy was also validated (16, 17). Reportedly, CYFRA21-1 was significantly and positively correlated with esophageal cancer regression, tumor invasion depth and TNM stage (16, 18). We obtained similar results. The pretreatment positive rate of CYFRA21-1 was correlated with gender, tumor location, T stage, N stage and TNM stage. The positive rate of CYFRA21-1 was higher at deeper tumor invasion and later stage (all P < 0.05) and was higher at higher N stage (Fisher = 12.25, P = 0.003), which are consistent with another study (17). In terms of genders, the positive rate of CYFRA21-1 in male patients was higher than in female patients (X^2^ = 8.05, P = 0.005). In addition, CYFRA21-1 level increased significantly in the recurrent group, which is consistent with previous research that the CYFRA21-1 level elevation in ESCC patients is related to the poor prognosis caused by tumor progression (19), and reflects its role in the monitoring of esophageal cancer recurrence. Furthermore, the experiments on the changes of CYFRA21-1 level after treatment of esophageal cancer found that the positive rate of CYFRA21-1 significantly declined after treatment (P < 0.05), and the changes are consistent with the clinical efficacy.

NSE is an acidic protease expressed in neural tissues, and a specific marker of neuroendocrine tumors. Since 80% of lung cancer cases are neuroendocrine derived tumors, NSE is considered to be a highly sensitive tumor marker of lung cancer (20–22). It also plays an important role in early prediction of lymph node metastasis of lung cancer (23). However, the significance of NSE in esophageal cancer is not much concerned yet. As we know, this is the first time to study the values of NSE in the diagnosis and efficacy prediction of radical radiotherapy and chemotherapy for esophageal cancer. Results show NSE has important value in the diagnosis, short-term efficacy evaluation and recurrence monitoring of esophageal cancer. Moreover, NSE level before treatment is significantly higher than that in the control group. NSE level is significantly reduced after treatment (P<0.05). The serum NSE levels of recurrent patients also significantly rose, reflecting its role in the monitoring of esophageal cancer recurrence. There are few studies on the values of NSE in esophageal cancer. This study demonstrates that the clinical CR or PR rate of pretreatment NSE-positive patients is significantly lower after radiotherapy and chemotherapy compared with pretreatment NSE-negative patients, and the short-term curative effect is poor. Similarly, NSE value is positively correlated with the load of Merkel cell carcinoma (24). Furthermore, small cell lung cancer patients with high NSE level have poor prognosis (25). Although it is concluded that both positive and negative NSE affects the short-term curative effect of patients and is related to the early recurrence, NSE content is not significantly related to clinical stage, tumor location, or T, N or TNM stage at the clinicopathological level, which is inconsistent with other research (24). The inconsistency may be related to the cut-off value of NSE. In addition, our sample size is relatively small, which needs to be verified by further large-sample research. When the routine cut-off value of NSE is adopted, the positive rate of NSE in the serum of the control group is also high (42%), the cut-off value of NSE with the most diagnostic efficacy in ESCC is 17.5 ng/ml and the area of AUC is 0.64 (P < 0.05). However, the best cut-off value was not obtained in predicting the curative effect, which still needs to be verified by a large-sample prospective study.

In fact, due to the low sensitivity, it is difficult to use a single positive tumor marker for diagnosis and prognosis (26). In this study, CEA, CYFRA21-1 and NSE all significantly changed in the diagnosis of esophageal cancer. The value of these tumor markers in the diagnosis of esophageal cancer was further verified by ROC curves. The sensitivity and specificity of the three markers were 80%, 88.89%, 58.5% and 53%, 60% and 58% respectively. The AUC areas were 0.70, 0.70 and 0.64 respectively (all P < 0.05). With the combined detection, the sensitivity and specificity were 68% and 78% respectively. In terms of prognosis prediction, CYFRA21-1 and NSE are better. Therefore, to more accurately evaluate the prognostic values of these two markers, the levels of serum NSE and CYFRA21-1 may be combined to improve the sensitivity, which will become a useful predictor. After comparing the TM(+) rates before treatment with a recent efficacy study, we found a significant correlation between the serum TM(+) rate before treatment and the efficacy (P = 0.03). The CR + PR rate in the TM(+) group was significantly lower than that in the TM(-) group. With a higher TM (+) rate before treatment, the probability of patients to reach clinical CR/PR was lower and the prognosis was worse, which is consistent with previous studies (27, 28). Therefore, we believe that ESCC with higher TM(+) rate is less sensitive to radiotherapy and chemotherapy. However, this conclusion should be confirmed by further studies with larger and more uniform samples. The detection of serum NSE and CYFRA21-1 levels is cheap, noninvasive and suitable for routine clinical use. Although the use of more sensitive SCCmRNA and other methods to predict the prognosis of esophageal cancer was investigated (29), this molecular detection method is very expensive and cannot be used routinely in clinic. Our results show that the short-term clinical efficacy of ESCC patients based on serum TM(+) is worse than that of TM(-). TM(+) combined with serum NSE and CYFRA21-1 can be used as a new variable to predict the prognosis of ESCC patients.

Previous articles also focused on other prognostic biomarkers for ESCC, such as SCC-Ag and HER-2 (30, 31). An earlier published study showed that high preoperative SCC-Ag concentrations (> 1.1 ng/ml), which were significantly associated with more aggressive tumor phenotypes and shorter disease-free survival, were identified as an independent prognostic factor in the multivariable analysis (30). Reportedly, the expressions of HER-2 mRNA was related to the lymph node metastasis in ESCC and pathologic differentiation degree (32).

There are still some limitations in the current study. First of all, this study is based on a single treatment center, which may have bias and thus calls for multicenter collaborative research to verify our results. Secondly, this retrospective study involves a small number of patients, so the results still need further verification. At last, no long-term follow-up was conducted. The effects of the pretreatment positive rates of tumor markers on the overall survival and progression-free survival of esophageal cancer patients are unknown.

## Conclusion

The values of CYFRA21-1 and NSE hematological indexes before CRT in the diagnosis and short-term efficacy monitoring of ESCC were studied. CEA, CYFRA21-1 and NSE have certain values in the diagnosis of esophageal cancer, while CYFRA21-1 and NSE have obvious values and can be combined in the efficacy evaluation and recurrence monitoring of ESCC. However, unconventional cut-off values may limit the interpretability and extrapolation of the research results. We expect large-sample prospective studies to further evaluate the application values and clinical significance of CYFRA 21-1 and NSE in ESCC patients.

## Data Availability Statement

The datasets presented in this study can be found in online repositories. The names of the repository/repositories and accession number(s) can be found in the article/supplementary material.

## Ethics Statement

The studies involving human participants were reviewed and approved by Jurong People’s Hospital Ethics Committee. The patients/participants provided their written informed consent to participate in this study. Written informed consent was obtained from the individual(s) for the publication of any potentially identifiable images or data included in this article.

## Author Contributions

MJ contributed to the conceptualization and editing. YS contributed to the literature search, data analysis, case data sorting review. XG contributed to provide radiotherapy and chemotherapy cases of esophageal cancer and revision. XD contributed to the literature search and editing. YZ contributed to the modification of figures. LL contributed to revision and data analysis. YS contributed to funding acquisition. All authors have read and approved the final manuscript.

## Funding

This work was supported by the Jurong Min sheng science and technology project of China (grant 2020SA00106),Jiangsu Science and technology project (grant BK20201080).

## Conflict of Interest

The authors declare that the research was conducted in the absence of any commercial or financial relationships that could be construed as a potential conflict of interest.

## Publisher’s Note

All claims expressed in this article are solely those of the authors and do not necessarily represent those of their affiliated organizations, or those of the publisher, the editors and the reviewers. Any product that may be evaluated in this article, or claim that may be made by its manufacturer, is not guaranteed or endorsed by the publisher.

## References

[1] Bray F Ferlay J Soerjomataram I Siegel RL Torre LA Jemal A. Global Cancer Statistics 2018: GLOBOCAN Estimates of Incidence and Mortality Worldwide for 36 Cancers in 185 Countries. CA Cancer J Clin (2018) 68:394–424. doi: 10.3322/caac.21492 30207593

[2] Feng RM Zong YN Cao SM Xu RH. Current Cancer Situation in China: Good or Bad News From the 2018 Global Cancer Statistics? Canc Commun (2019) 39:22. doi: 10.1186/s40880-019-0368-6 PMC6487510 31030667

[3] Shu J Li CG Liu YC Yan XC Xu X Huang XE. Comparison of Serum Tumor Associated Material (TAM) With Conventional Biomarkers in Cancer Patients. Asian Pac J Cancer Prev (2012) 13(5):2399–403. doi: 10.7314/apjcp.2012.13.5.2399 22901228

[4] Yan HJ Wang RB Zhu KL Jiang SM Zhao W Xu XQ. Cytokeratin-19 Fragment Antigen 21-1 as an Independent Predictor for Definitive Chemoradiotherapy Sensitivity in Esophageal Squamous Cell Carcinoma. Chin Med J (Eng 1) (2012) 125(8):1410–5. doi: 10.3760/cma.j.issn.0366-6999.2012.08.009 22613644

[5] Zhang Y Huang J Zou Q Che J Yang K Fan Q. Methylated PTGER4 is Better Than CA125, CEA, Cyfra211 and NSE as a Therapeutic Response Assessment Marker in Stage IV Lung Cancer. Oncol Lett (2020) 19(4):3229– 3238. doi: 10.3892/ol.2020.11434 32256818 PMC7074558

[6] Bates SE. Clinical Applications of Serum Tumor Markers. Ann Intern Med (1991) 115:623–38. doi: 10.7326/0003-4819-115-8-623 1716430

[7] McKnight A Mannell A Shperling I. The Role of Carbohydrate Antigen 19-9 as a Tumor Marker of Oesophageal Cancer. Br J Cancer (1989) 60:249–51. doi: 10.1038/bjc.1989.263 PMC2247033 2765375

[8] Nakamura T Ide H Eguchi R Hayashi K Takasaki K Watanabe S. CYFRA 21-1 as a Tumor Marker for Squamous Cell Carcinoma of the Esophagus. Dis Esophagus (2017) 11(1):35–9. doi: 10.1093/dote/11.1.35 29040480

[9] Kosugi S Nishimaki T Kanda T Nakagawa S Ohashi M Hatakeyama K. Clinical Significance of Serum Carcinoembryonic Antigen, Carbohydrate Antigen 19-9, and Squamous Cell Carcinoma Antigen Levels in Esophageal Cancer Patients. World J Surg (2004) 28(7):680–5. doi: 10.1007/s00268-004-6865-y 15383868

[10] Mroczko B Kozłowski M Groblewska M Łukaszewicz M Nikliński J Jelski W. The Diagnostic Value of the Measurement of Matrix Metalloproteinase 9 (MMP-9), Squamous Cell Cancer Antigen (SCC) and Carcinoembryonic Antigen (CEA) in the Sera of Esophageal Cancer Patients. Clin Chim Acta (2008) 389:61–6. doi: 10.1016/j.cca.2007.11.023 18155162

[11] Clark GW Ireland AP Hagen JA Collard JM Peters JH DeMeester TR. Carcinoembryonic Antigen Measurements in the Management of Esophageal Cancer: An Indicator of Subclinical Recurrence. Am J Surg (1995) 170:597–600. doi: 10.1016/S0002-9610(99)80023-3 7492008

[12] Liu L Liu B Zhu LL Li Y. CYFRA21-1 as a Serum Tumor Marker for Follow-Up Patients With Squamous Cell Lung Carcinoma and Oropharynx Squamous Cell Carcinoma. biomark Med (2013) 7(4):591–9. doi: 10.2217/bmm.13.55 23905896

[13] Pujol JL Grenier J Daurès JP Daver A Pujol H Michel FB. Serum Fragment of Cytokeratin Subunit 19 Measured by CYFRA 21-1 Immunoradiometric Assay as a Marker of Lung Cancer. Cancer Res (1993) 53:61–6. 7677981

[14] Stieber P Hasholzner U Bodenmüller H Nagel D Sunder-Plassmann L Dienemann H. CYFRA 21-1. A New Marker in Lung Cancer. Cancer (1993) 72:707–13. doi: 10.1002/1097-0142(19930801)72:3<707::AID-CNCR2820720313>3.0.CO;2-X 7687515

[15] Yuan C Yang K Tang H Chen D. Diagnostic Values of Serum Tumor Markers Cyfra21-1, SCCAg, Ferritin, CEA, CA19-9, and AFP in Oral/Oropharyngeal Squamous Cell Carcinoma. Onco Targets Ther (2016) 9:3381–6. doi: 10.2147/OTT.S105672 PMC4902246 27350753

[16] Yamamoto K Oka M Hayashi H Tangoku A Gondo T Suzuki T. CYFRA 21-1 is a Useful Marker for Esophageal Squamous Cell Carcinoma. Cancer (1997) 79:1647–55. doi: 10.1002/(SICI)1097-0142(19970501)79:9<1647::AID-CNCR3>3.0.CO;2-9 9128978

[17] Shimada H Nabeya Y Okazumi S Matsubara H Miyazawa Y Shiratori T. Prognostic Significance of CYFRA 21-1 in Patients With Esophageal Squamous Cell Carcinoma. J Am Coll Surg (2003) 196(4):573–8. doi: 10.1016/S1072-7515(02)01905-1 12691934

[18] Shimada H Nabeya Y Okazumi S Matsubara H Miyazawa Y Shiratori T. Prognostic Signifificance of CYFRA 21-1 in Patients With Esophageal Squamous Cell Carcinoma. J Am Coll Surg (2003) 196:573–8. doi: 10.1016/S1072-7515(02)01905-1 12691934

[19] Qiao Y Chen C Yue J Yu Z. Tumor Marker Index Based on Preoperative SCC and CYFRA 21-1 Is a Significant Prognostic Factor for Patients With Resectable Esophageal Squamous Cell Carcinoma. Cancer biomark (2019) 25:243–50. doi: 10.3233/CBM-190058 PMC13082431 31282406

[20] Korkmaz ET Koksal D Aksu F Dikmen ZG Icen D maden E. Triple Test With Tumor Markers CYFRA 21.1, HE4, and ProGRP Might Contribute to Diagnosis and Subtyping of Lung Cancer. Clin Biochem (2018) 58:15–9. doi: 10.1016/j.clinbiochem.2018.05.001 29729229

[21] Jiang ZF Wang M Xu JL. Thymidine Kinase 1 Combined With CEA, CYFRA21-1 and NSE Improved its Diagnostic Value for Lung Cancer. Life Sci (2018) 194:1–6. doi: 10.1016/j.lfs.2017.12.020 29247745

[22] Shirasawa M Fukui T Kusuhara S Hiyoshi Y Ishihara M Kasajima M. Prognostic Significance of the 8th Edition of the TNM Classification for Patients With Extensive Disease Small Cell Lung Cancer. Cancer Manag Res (2018) 10:6039–47. doi: 10.2147/CMAR.S181789 PMC6252783 30538553

[23] Li Y Kuang Y Jia Y Bai S. Diagnostic Value of NSE Factor Combined With Ultrasound Hemodynamic Indexes in Cervical Lymph Node Metastasis of Lung Cancer. Oncol Lett (2020) 20(1):699–704. doi: 10.3892/ol.2020.11621 32565995 PMC7285818

[24] van Veenendaal LM Bertolli E Korse CM Klop WMC Tesselaar MET van Akkooi ACJ. The Clinical Utility of Neuron-Specific Enolase (NSE) Serum Levels as a Biomarker for Merkel Cell Carcinoma (MCC). Ann Surg Oncol (2021) 28(2):1019– 1028. doi: 10.1245/s10434-020-08656-7 32529274

[25] Pinson P Joos G Watripont P Brussell G Pauwels R. Serum Neuron-Specific Enolase as a Tumor Maker in the Diagnosis and Follow-Up of Small-Cell Lung Cancer. Respiration (1997) 64:102–7. doi: 10.1159/000196651 9044484

[26] Daver A Dalifard I Pons-Anicet D Krebs BP Gosselin P Cazin JL. Diagnostic Value of SCC-TA4 Determination in 4 Localizations of Epidermoid Cancers. An Experience of the FNCLCC Subgroup of Radio-Analysis. Bull Cancer (1990) 77:781–92. A. Et. 2207367

[27] Yi Y Li B Wang Z Sun H Gong H Zhang Z. CYFRA21-1 and CEA are Useful Markers for Predicting the Sensitivity to Chemoradiotherapy of Esophageal Squamous Cell Carcinoma. Biomarkers (2009) 14(7):480–5. doi: 10.3109/13547500903180265 19863186

[28] Wakatsuki M Suzuki Y Nakamoto S Ohno T Ishikawa H Kiyohara H. Clinical Usefulness of CYFRA 21-1 for Esophageal Squamous Cell Carcinoma in Radiation Therapy. J Gastroenterol Hepatol (2007) 22:715–19. doi: 10.1111/j.1440-1746.2006.04498.x 17444861

[29] Qiao YF Chen CG Yue J Ma Z Yu ZT. Clinical Signifificance of Preoperative and Postoperative Cytokeratin 19 Messenger RNA Level in Peripheral Blood of Esophageal Cancer Patients. Dis Esophagus (2016) 29:929–36. doi: 10.1111/dote.12403 26382739

[30] Kanda M Koike M Shimizu D Tanaka C Kobayashi D Hattori N. Optimized Cutoff Value of Serum Squamous Cell Carcinoma Antigen Concentration Accurately Predicts Recurrence After Curative Resection of Squamous Cell Carcinoma of the Esophagus. Ann Surg Oncol (2020) 27(4):1233–40. doi: 10.1245/s10434-019-07977-6 31650302

[31] Heidarpour M Taheri M Akhavan A Goli P Kefayat A. Investigation of HER-2 Expression an Its Correlation With Clinicopathological Parameters and Overall Survival of Esophageal Squamous Cell Carcinoma Patients. Iranian J Pathol (2020) 15(4):274–81. doi: 10.30699/ijp.2020.113829.2235 PMC7477677 32944039

[32] Zhang L Wang Y Bai G Zhang J Yang M Ma X. The Relationship Between the Expression of VEGF, EGFR, and HER-2 mRNA in Esophageal Squamous Cell Carcinoma (ESCC) and Clinicopathological Features of Different Ethnic Groups in Xinjiang. Tumour Biol (2015) 36(12):9277–83. doi: 10.1007/s13277-015-3656-z PMC4689756 26099724

